# Extraction of grape seeds by different solvents affects the activities of the resultant extract

**DOI:** 10.1186/s13568-025-01851-3

**Published:** 2025-03-19

**Authors:** Kareem Tarek, Alyaa Farid, Gehan Safwat

**Affiliations:** 1 Biotechnology Department, Faculty of Science, Cairo University, Giza, Egypt; 2 Faculty of Biotechnology, October University for Modern Sciences and Arts, Giza, Egypt

**Keywords:** Grape seed, Ethanol, Methanol, Flavonoids, Phenolic compounds

## Abstract

Phenolic compounds are concentrated in grape seeds; 60–70% of the extractable grape phenols are found in the seeds. The focus of this research was to isolate the phytochemicals from grape seed and to determine their ability to prevent haemolysis, their antioxidant and microbiological activities. By using the extraction procedure, three solvents were used (distilled water, ethanol and methanol). A high-performance liquid chromatographic (HPLC) test was performed to analyse the phenolic compounds and flavonoids content that were used to determine the efficiency of the various solvents used in the extraction process. All the variables under study, namely yield percentage, phenolic component concentration, and flavonoid content got significantly affected by the choice of the solvent used. The flavonoid content of the extracts was in the order methanolic extract > ethanolic extract > water extract. The methanolic extract of the grape seeds exhibited the most powerful antioxidant and hemolysis inhibitory effects among the three extracts, followed by the ethanolic and water extracts. The antibacterial activity of methanolic extract was found to be higher as compared to the ethanolic extract against *Staphylococcus aureus*. The antibacterial activity of the ethanolic and methanolic extracts against *Salmonella enteritidis*, *Bacillus subtilis*, *Aspergillus niger* and *Escherichia coli* were found to be equivalent. In conclusion, grape seeds contained several bioactive compounds that exerted an antioxidant, hemolysis inhibition and anti-microbial activities. These activities depends on the concentration of phenolic compounds and flavonoids in the grape seed extracts. Methanol was the superior solvent in the extraction process followed by ethanol.

## Introduction

Industries generate high percent (30%) of waste products during the processing of fruits and vegetables (Mahato et al. 2019). Processing companies yield a number of important by-products, such as peels, seeds, foliage and roots. The environment is impacted by the disposal of these waste products, and industries impacted economically (Kapoor et al. 2020). However, these waste products constitute a great source of bioactive components like essential oils, fibers, biologically active amino acids, and polyphenols (phenolic compounds and flavonoids). Fruit seeds, for example, offer an excellent way to obtain phytochemical compounds, phytosteroids, and essential oils. The pectin that is an important fibers is found in the fruit peels in addition to several minerals (Marić et al. 2018; Mena-García et al. 2019).

The seeds of grape (*Vitis vinifera* L.) used to be categorized as by-products, from grape juice and wine industries, that can be best utilized in the food industry (Molva and Baysal 2015). It has many health benefits due to its content of proteins, fibers, oils, carbohydrates, polyphenols, vitamins and minerals as noted by Mayer et al. (2008). According to Thorngate and Singleton (1994), the average distribution of polyphenols concentration in grapes is approximately five percent in the juice, one percent in the pulp, and approximately sixty two percent of the remaining amount in the seeds. The majority of the polyphenols found in grape seeds are flavonoids, such as epicatechins, gallocatechins, epigallocatechines, epicatechin 3–0 gallate, monomeric flavan-3-ols catechins, gallic acid and procyanidins (dimmer, trimers and polymerized) (Prieur et al. 1994). These chemicals are now recognized for their potent antioxidant, wrinkle prevention, anti-inflammatory, anti-cancer, ulcer healing, anti-bacterial and non-atherogenic properties (Amellal et al. 1985; Liviero and Pulglisi 1994; Saito et al. 1998b, a; Palma et al. 1999; Caterall et al*.* 2000; Del Bas et al. 2005). It was discovered that grape seed extract plays a major role in the medical field because it has antioxidant, anti-inflammatory, antidiabetic, hepatoprotective, cardioprotective and renoprotective effects. These effects have been found to eliminate many human diseases as mentioned by Daus (2007). In addition, due to the high content of the 3, 4, 5-trihydroxyphenyl groups in tannins and catechins, the grape seed extracts have received a lot of attention as an antibacterial agent (Taguri et al. 2004).

The Food and Drug Administration (FDA) has approved grape seed extract as a GRAS (Generally Recognized as Safe) nutritional supplement that is sold widely. Few studies, however, have shown the negative effects of grape seed extract, such as its anticoagulant effects and interactions with other medications, whether taken either by itself or in conjunction with others (Rein et al. 2000; Shanmuganayagam et al. 2002). While grape seed extract is utilized at relatively low concentrations (0.01–1%) in food applications, it may be useful when used at pharmaceutical levels of 0.15–0.3 g/day (Clouatre and Kandaswami 2005). Additionally, 1.78 g/kg b. wt/day was the NOAEL (No Observed-Adverse Effect Level) of the grape seed extract found in the rats, which was clearly a greater concentration level than it is often employed in food applications (Bentivegna and Whitney 2002).

Numerous studies have demonstrated that grape seeds extract can strengthen the antioxidant defense mechanisms, including preventing liver damage from carbon tetrachloride and ischemia/reperfusion (Dai et al. 2014; Xu et al. 2015), reducing arsenic-induced oxidative reproductive toxicity, and protecting against cisplatin and amikacin-induced kidney injury (Gao et al. 2014). Grape seed extract prevents atherosclerosis through a variety of ways such as limiting low-density lipoprotein oxidation, reducing blood pressure, minimizing inflammation, and inhibiting platelet aggregation (Dohadwala and Vita 2009). Consuming diets high in phenolic antioxidants decreased the incidence of heart disease, and the in vitro and in vivo studies showed a correlation between the presence of antioxidant phenolics and the decrease of oxidized low-density lipoproteins (Kinsella et al. 1993). The prevention of cardiovascular disease is directly related to the grape seeds polyphenols' ability to prevent the radical oxidation of polyunsaturated fatty acids (in low-density lipoproteins), which frequently happens as a result of oxidative transformation of the apoprotein to an atherogenic form (Rimm et al. 1993). When grape seeds' antihypertensive properties were examined, it was discovered that their proanthocyanidin-rich extract can effectively lower blood pressure by suppressing oxidative stress, blocking the angiotensin converting enzyme, and modulating nitric oxide-induced blood vessel dilation (Gonzalez et al. 2014). Through the suppression of lipid oxidation and the microviscosity of the gastrointestinal membranes, grape seed extract rich in proanthocyanidin has been shown to promote immunity against both acute and chronic gastrointestinal oxidative stress (Bagchi et al. 1999) and demonstrated greater gastroprotective properties when compared to vitamins C and E (Cuevas et al. 2011). The capacity of grape seeds' procyanidins to bind proteins on the gastrointestinal surface was the cause of this gastroprotective activity (Saito et al. 1998b, a). Phenolic chemicals found in grape seeds showed antitumor and cell cycle modulating activity (Huang et al. 2012). According to Engelbrecht et al. (2007), these phenolic compounds exhibit cytotoxic capabilities towards cancer cells while having no effect on healthy normal cells. The deactivation of the signaling pathway of phosphoinositide 3-kinase/protein kinase B (triggers cancer cell apoptosis), and the expression of the pro-angiogenic factors (angiopoietins and the vascular endothelial growth factor) are the proposed mechanisms for the extract cancer-fighting properties (Engelbrecht et al. 2007; Huang et al. 2012). According to Jayaprakasha et al. (2003a, b), grape seed extract has demonstrated an effective antimicrobial property. It works well against gram-positive bacteria such as *Bacillus subtilis* and *Staphylococcus aureus*, but it works better against gram-negative bacteria such as *Pseudomonas aeruginosa* and *Escherichia coli*. Proanthocyanidin-rich grape seed extract suppresses the generation of free radicals and lowers lipid peroxidation of the skin's cellular structure (Markus and Morris 2008). Grape seeds extract is a common element in a lot of products that are on the market. These products consist of tablets, pills, creams, lotions, and more. Other active ingredients in the extract include phenolic acids, phytosterol, flavonoids, tocopherol, carotenoids, and tocotrienol, which have strong antioxidant properties and can support healthy skin (Devi and Singh 2017).

There are various technologies that can be employed to recover the bioactive compounds from the plant’s waste products and employ them in the production of other valuable products like nutritional supplements. Besides, this helps in reducing the volume of waste that is dumped into the environment (Patra et al. 2022). The method that is used in the extraction procedure depends on the type of extract purity required, the properties of the target compound and the state of the compound whether it is in free form or enclosed in cells, the value and cost of the derived product. For a higher yield, plant materials are washed, grinded to reduce the particle size and dried prior to the extraction process (Gong et al. 2020). The primary goals of the extraction process are to obtain the desired bioactive substances from complicated samples, improve the analytical methods' selectivity, boost the sensitiveness of biological test by raising the amount of the desired substances, transform the bioactive substances into a form better suited for separation and detection and offer a robust, repeatable procedure that is unaffected by changes in the sample (Azmir et al. 2013). The fundamental principles of solvent extraction procedure involve the penetration of solvent into dried plant sample, dissolving of bioactive materials and their diffusion from the sample and the collection of the extracted components (Smith 2003).

Thilakarathna and Rupasinghe (2022) developed an ethyl alcohol-based method to extract proanthocyanidins from grape seeds. Dabetic et al. (2022) optimized the extraction of polyphenols from red grape seeds by conventional deep eutectic solvents. Moutinho et al. (2023) reported that grape seeds extracted at 60 °C with HCl (3.5%) and ethanol (70%) had the best yield and antioxidant activity. There were 44.93 mg of gallic acid equivalents/g and 22.95 mg of catechin equivalents/g for total phenols and flavonoids, respectively. Several parameters influence the extraction of polyphenols from grape seeds like the type of solvent, heat, solid to liquid percentage, duration of extraction process, technique used, the storage conditions, and the existence of competing chemicals. Furthermore, because plant-derived polyphenols differ greatly in their chemical structure, it is practically hard to create an extraction technique that works for all phenolic compounds (Robards 2003a, b; Naczk and Shahidi 2004). The selection of solvent constitutes the most important aspects in the method of extraction since the polyphenols solubility and how they diffuse to the solvent rely on their chemical composition, which from basic to heavily formed polymers. The most commonly utilized solvents, with varying degrees of efficacy, for the extraction of plant polyphenols comprise alcohols (ethanol, methanol and propanol), ketone (acetone), ester (ethyl acetate), formamides (dimethylformamide), in addition to their mixture and their combination with water (Escribano-Bailon and Santos-Buelga 2003).

Because various solvents react differentially with specific elements of a natural substance, the extraction solvent used has an impact on the bioactivity of the plant materials (Kavaz and Ogbonna 2019). Although ethanol is safer to be used than methanol, methanol has been shown to be a better solvent for extracting bioactive and antioxidant-rich compounds (Rahman et al. 2018). In solvent extraction for phytochemical research, alcohols (ethanol and methanol) represent common solvents (Zhang et al. 2018). Independent of the extraction technique used, alcohols were found to be the most effective solvents for extracting compounds having antioxidant and antibacterial properties (Borges et al. 2020). According to Ermi Hikmawanti et al. (2021), ethanol is a solvent that is acceptable for human ingestion when used to dissolve natural ingredients for use in food and medicine. According to Sultana et al. (2009), polyphenolic derived antioxidant chemicals have been effectively extracted from natural substances using both absolute and aqueous ethanol. Methanol's capacity to effectively extract a broad variety of phytochemical components from plant material is the basis for its usage in the production of plant extracts in the drug design industry. Because it can solubilize both polar and non-polar molecules, methanol is a frequently used solvent in laboratory settings and can be used to extract a wide range of plant elements. Because of its increased volatility, lower boiling point, and greater processing efficiency (based on the favored secondary metabolite compositions), methanol is employed to extract medicinal plants. When compared to other extracts, the methanolic extract has demonstrated higher flavonoid levels. Thus, the most effective solvent for removing flavonoids and phenolic chemicals from therapeutic plants is methanol (Ghanimi et al. 2022).

The present study aimed to extract the bioactive components from grape seeds and test their antioxidant, antimicrobial and hemolysis inhibition effects. Three solvents (distilled water, ethanol and methanol) were used in the extraction process. The grape seed extractions were analyzed by high performance liquid chromatography (HPLC) to identify their phenolic compounds and flavonoids. The efficacy of the different solvents (distilled water, ethanol and methanol) in the extraction process was evaluated from the yield percent and the concentration of the extracted phenolic compounds and flavonoids. Furthermore, the antioxidant and antimicrobial activities of each extract were used in determining the efficacy of each solvent.

## Materials and methods

### Preparation of grape seeds extract

Muscat of Alexandria grape seeds were washed by distilled water and allowed to dry in the shade at room temperature then kept at 40°C in the oven for ten hours. Dried seeds were grinded into a fine powder by the aid of a mortar then sieved by a mesh. Grape seed powder (100 g) was mixed with 360 ml solvent [distilled water, ≥ 95.0% absolute ethanol (493511, Sigma-Aldrich, USA) or absolute methanol (acetone free, M1775, Sigma-Aldrich, USA)]; and kept in dark bottles at room temperature for 24 h. Different grape seeds extracts were filtered to obtain a clear filtrate which was evaporated at 30 °C by a rotary evaporator; then converted into powder by the aid of a lyophilizer.

### Proximate analysis of grape seeds

The Association of Official Analytical Chemists (A.O.A.C) methods were used to determine the fat, ash, moisture, protein and crude fiber content in grape seeds (Lee et al. 1992). The tests were repeated three times and the data were expressed as mean ± standard deviation (SD). Kjeldahl method was used to determine the crude protein content. This method based on the conversion of nitrogen in grape seeds into ammonium sulfate by digestion with H_2_SO_4_ followed by alkalization with NaOH to form ammonia. Ammonia was distilled in known volume of a standard acid. The residual acid, represented the nitrogen content in the sample, was determined by titration. Total protein content = total nitrogen content × 6.25).

For fat content determination, AOAC method 960.39 was used (AOAC 2002). Five g of grape seeds powder were put into a cellulose thimble and extracted with hexane [Certified, Fisher Chemical, catalogue no. H291-500) for 30 min at 70 °C. After extraction, the solvent was evaporated at low temperature in an operating fume hood. The extraction cups were dried and the fat content was determined from the next equation: crude fat content = weight of cup with fat residue-weight of empty cup.

Carbohydrate content was determined from the formula (Felhi et al. 2016): Carbohydrate content = dry weight (100%) − total sum of fat, protein, ash and moisture.

For ash content determination, four gram of grape seeds powder was ashed in a porcelain crucible at 500 °C for 6 h. For determination of metals and minerals the atomic absorption spectroscopy was used. Dried grape seeds powder (2 g) was ashed in a porcelain crucible at high temperature (550 °C). The formed ash was dissolved in diluted HCl (1 ml of concentrated HCl mixed with 100 ml distilled H_2_O) then filtered. The resulted ash was used in the determination of different elements. The energy was determined from (Felhi et al. 2016):

Value of energy (kcal) = 4 × protein content (g) + 4 × carbohydrate content (g) + 9 × fat content (g).

### Estimation of the total phenolic compounds content (Folin–Ciocalteu method)

The Julkunen-Tiitto (1985) method was used to determine the total phenolic content in prepared grape seeds extracts. Each grape seed extract (0.05 ml) was mixed, individually, with 1.95 ml distilled water and one ml of Folin–Ciocalteu reagent; and the mixtures were agitated vigorously. Five ml of a Na_2_CO_3_ solution (20%) was added and the mixture's volume was increased to 10 ml. The mixtures were agitated again for 15 min and the absorbance was measured at 735 nm. The gallic acid standard curve was used to quantify the total phenolic content of the extracts; and the results were calculated as milligrams of gallic acid equivalents/100 g of dry weight.

### Estimation of the total flavonoids content (AlCl_3_ colorimetric assay)

The AlCl_3_ colorimetric method was utilized to measure the total flavonoids content in grape seeds extracts (Huang et al. 2006). Various grape seeds extracts (one ml) containing different concentrations were combined, individually, with two ml methanolic AlCl_3_·6H_2_O (2%). The mixtures were allowed to stand for 15 min and their absorbance was read at 430 nm wave length. According to quercetin calibration curve, total flavonoids content was calculated and expressed as milligrams of quercetin per 100 g of dry matter.

### Analysis of grape seeds extracts by high performance liquid chromatography (HPLC)

A 0.45 µm filter was used to filter all grape seed extracts prior to their injection into the HPLC apparatus (Bucić-Kojić et al. 2009). The analysis was carried out using Beckman C18 column (4.6 × 100 mm, particles size of 3.5µm) which was eluted at 1 ml/min by an autosampler at 20°C. Two solvents were used in the process which were: A = acetic acid/water and B = acetic acid/methanol. The elution conditions were as follows: 0–30 min from 5 to 80% B; 30–33 min from 80% B; and 33–35 min from 80 to 5% B. Standard and extract injection volumes were 20 µl. Individual compounds were identified by comparing their spectral data (280 nm) and retention durations with standards.

### Determination of the antioxidant activity (DPPH scavenging assay)

The antioxidant activities of the different grape seeds extracts were determined by spectrophotometric method against stable 2.2-diphenyl-1-picrylhydrazyl radical (DPPH) according to Sanchez-Moreno et al. (1998). Grape seed extracts (0.1 ml) was mixed, individually, with 3.9 ml of DPPH (6.6 × 10^–5^ mol/l) prepared by dissolving 2.6 mg of DPPH in methanol (100 ml) and allowed to stand in the dark for half an hour. The absorbance was measured at 515 nm and the data was expressed in µMol/g of Trolox equivalent antioxidant capacity (TEAC). The test was repeated three times and the data were expressed as mean ± standard deviation (SD).

### Determination of the hemolytic activity (hemolysis inhibition test)

Erythrocytes suspension was prepared from rat's blood Nasser et al. (), where fresh heparinized blood sample (5 ml) was centrifuged (1000 rpm and 10 min) and the resultant pellet was washed with equal volume of phosphate buffered saline (PBS) until a colourless supernatant appeared. The pellet was mixed with 2 ml PBS (pH = 7) to form an erythrocyte suspension (4%). Two hundreds μl of grape seeds extracts at different concentrations (100, 200, 400, 600, 800 and 1000 μg/ml) were mixed, individually, with 150 μl of prepared erythrocytes suspension. The mixtures were incubated at 37 °C for an hour then centrifuged at 1000 rpm for 10 min. The released haemoglobin in the supernatant was measured at 540 nm. The result of tested extracts was normalized with respect to positive control (water) and a negative control (PBS). The test was repeated three times and the data were expressed as mean ± standard deviation (SD).

### Determination of the antimicrobial activity (disc inhibition zone)

The bacteria and fungi used in this study included the following gram-positive bacteria: *Bacillus subtilis* JN 934392 and *Staphylococcus aureus* ATCC 6538. It also included the following gram-negative bacteria: *Salmonella enteric* ATCC 43972 and *Escherichia coli* ATCC 25922. Additionally, fungal strains, such as *Aspergillus niger* and *Fusarium phyllophilum* AB587006, were tested. Mueller Hinton agar-containing Petri dishes were used to cultivate the tested bacteria, which were then incubated for 24 h. Bacterial culture was cultured in 3 ml Mueller Hinton broth with agitation at 200 rpm for 24 h at 37 °C (only, *Bacillus subtilis* was incubated at 30 °C). Fungal strains were grown on Sabouraud agar at 30 °C for 4 days. A spore suspension was then made in 10 ml of sterile water with 0.1% Tween 80. The determination of the inhibition zone diameter (IZ) was based on the same protocol as described by Daoud et al. (2019). Utilizing a sterilized Pasteur pipette, a cavity (6 mm) was made in the agar. A sterile swab was used to inoculate the agar surface with a freshly made 100 μl of bacterial suspension (10^7^ CFU/ml) or spore solution (10^6^ spores/ml). After that, 80 μl of each grape seeds extract (125 mg/ml deionized water) was added to each well. The plates were kept at + 4 °C for two hours to allow the extracts diffusion in the agar. The plate was incubated at 37 °C for 24 h (bacterial strains) or at 30 °C for 4–7 h (fungal strains). By measuring the diameter of the inhibitory zone surrounding the well, the antimicrobial activity was determined. The negative control was Mueller–Hinton broth and the positive control was gentamicin (100 µg/mL). Test was repeated three times and the data were expressed as mean ± standard deviation (SD).

### Statistical analysis

One-way analysis of variance (ANOVA) was used to determine the difference between different grape seeds extracts. The result was considered significance when *P* value was less than 0.05.

## Results

### Proximate analysis results

The proximate analysis revealed the presence of fat, protein and carbohydrates (2.86, 7.06 and 82.6 g/100 g, respectively) in the grape seeds that accounted for an energy of 384.46 kcal/100 g. The seeds contained 4.33 g/100 g ash and 3.23 g/100 g moisture (Table 1). High content of calcium and magnesium (41.76 and 67.90 mg/l) and low content of iron and zinc (4.13 and 1.66 mg/l) were found in the seeds; in addition to neglected content of mercury (0.002 mg/l) (Table 2). While cadmium was not detected in grape seeds, small concentrations of nickel (0.46 mg/l), copper (0.63 mg/l), lead (0.13 mg/l), manganese (0.23 mg/l) and sodium (0.83 mg/l) were detected.

**Table 1:** proximate analysis of grape seeds

Proximate analysis	Content
Fat (g/100 g)	2.86 ± 0.25
Protein (g/100 g)	7.06 ± 0.25
Total carbohydrates (g/100 g)	82.6 ± 0.43
Ash (g/100 g)	4.33 ± 0.25
Moisture (g/100 g)	3.23 ± 0.41
Energy (kcal/100 g)	384.46 ± 3.04

Results were expressed as mean ± SD, data obtained from three experiments

**Table 2:** Elements concentration in grape seeds

Elements	Concentration (mg/l)
Magnesium	67.90 ± 2.36
Iron	4.13 ± 0.15
Calcium	41.76 ± 1.65
Zinc	1.66 ± 0.15
Nickel	0.46 ± 0.20
Copper	0.63 ± 0.21
Lead	0.13 ± 0.05
Mercury	0.002 ± 0.001
Manganese	0.23 ± 0.15
Sodium	0.83 ± 0.25
Cadmium	ND

Results were expressed as mean ± SD, data obtained from three experiments

### Extraction yield

**Fig. 1:**
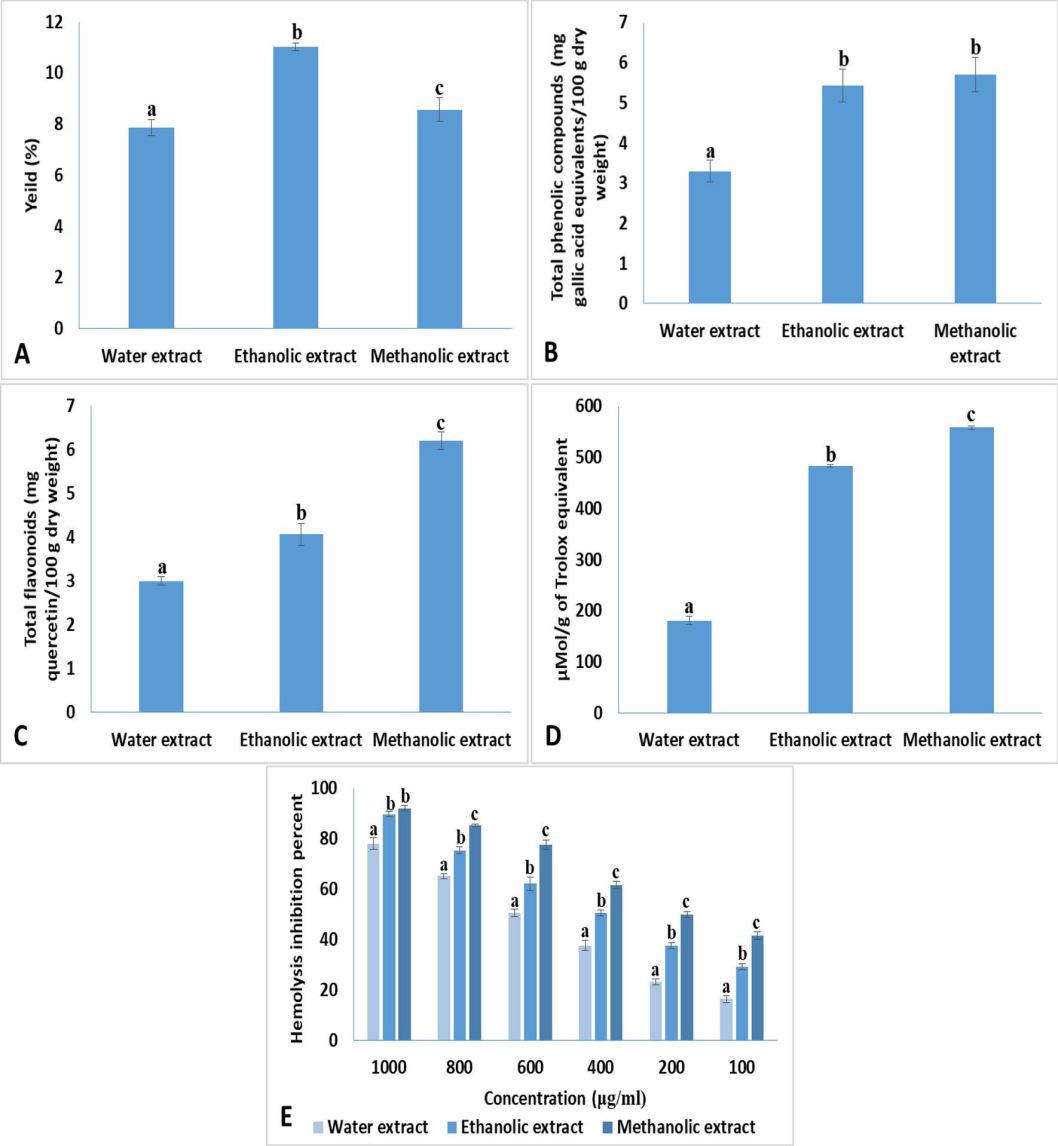
**A** Yield % of the extraction process, **B** total phenolic compounds obtained from different solvent (water, ethanol and methanol), **C** total flavonoids compounds obtained from different solvent (water, ethanol and methanol), **D** the antioxidant activity of different grape seed extracts and **E** the hemolysis inhibition activity of different grape seed extracts. Data with different letter were significantly different (*p* < 0.05)

The extraction yield was dependant on the solvent used in the extrcation process of grape seeds. Extraction of grape seeds with ethanol gave significantly the highest yield % (11.03%) followed by extraction with methanol (8.56%) then extraction with distilled water (7.86%) (Fig. 1A).

### Total phenolic compounds

No significance difference was observed in the total phenolic compounds content of ethanolic and methanolic grape seeds extracts (5.43 and 5.71 mg gallic acid equivalents/100 g dry weight, respectively). However, the lowest total phenolic compounds content was observed in the aqueous grape seeds extract (3.3 mg gallic acid equivalents/100 g dry weight) (Fig. 1B).

### Total flavonoids compounds

The highest flavonoids content was obtained from the methanolic grape seed extract (6.21 mg quercetin/100 g dry weight) followed by ethanolic extract (4.06 mg quercetin/100 g dry weight) then by water extract (3.00 mg quercetin/100 g dry weight) (Fig. 1C).

### The antioxidant activity

The antioxidant activity of the methanolic extract of grape seeds (558.30 µMol/g of Trolox equivalent) was significantly higher than that of the ethanolic extract (483.66 µMol/g of Trolox equivalent). On the other hand, the antioxidant activity of aqueous grape seed extract (180.96 µMol/g of Trolox equivalent) was significantly lower than those of the methanolic and ethanolic grape seeds extracts (Fig. 1D).

### The hemolysis inhibition activity

The hemolysis inhibition activity of the different grape seeds extracts was dose dependent (Fig. 1E). At a concentration of 1000 µg/ml, no significance difference was observed in the hemolysis inhibition % between methanolic and ethanolic grape seed extracts (92.13 and 89.76%, respectively). At the remaining tested concentrations (800, 600, 400, 200 and 100 µg/ml), the hemolysis inhibition activity of methanolic grape seeds extract was significantly higher than that of the ethanolic grape seeds extract. On the other hand, aqueous grape seeds extract showed a significant low hemolysis inhibition % when compared to ethanolic or methanolic grape seeds extract at all tested concentrations.

### The antimicrobial activity

Six microbial strains were used to test the antimicrobial activity of grape seeds extract. The three extracts showed significant inhibition zones that were recorded in Table 3. The three grape seeds extracts (water, ethanolic and methanolic extracts) showed an antimicrobial activity (inhibition zone) higher than those of positive control (gentamicin) and negative control (Mueller–Hinton broth). Water extract showed the largest inhibition zone for the fungal strains [*Aspergillus niger* (21.16 mm) and *Fusarium phyllophilum* (26.50 mm)] indicating its significant powerful antifungal activity when compared to ethanolic [*Aspergillus niger* (12.83 mm) and *Fusarium phyllophilum* (16.83 mm)] and methanolic [*Aspergillus niger* (11.16 mm) and *Fusarium phyllophilum* (13.50 mm)] extracts. Moreover, aqueous grape seed extract showed a significant higher antibacterial activity (inhibition zone) against *Escherichia coli* (14.50 mm) more than those of the ethanolic and methanolic grape seed extracts (10.83 and 11.96 mm, respectively). Ethanolic extract showed a significant high antibacterial activity (inhibition zone) against *Staphylococcus aureus* (18.83 mm) followed by the methanolic extract (15.83 mm) then the aqueous extract (10.66 mm). No significance difference was observed in the inhibition zone of the ethanolic and methanolic extracts against *Escherichia coli* (10.83 and 11.96 mm, respectively), *Salmonella enteritidis* (13.50 and 14.50 mm, respectively) and *Bacillus subtilis* (11.50 and 11.83 mm, respectively).

**Table 3:** The antimicrobial activity of grape seeds extracts

Microbes	Inhibition zone (mm)				
	Negative control (Mueller–Hinton broth)	Positive control (gentamicin)	Water extract	Ethanolic extract	Methanolic extract
Gram-negative bacteria					
* Escherichia coli*	2.53 ± 1.47^a^	9.06 ± 0.70^b^	14.50 ± 0.50^d^	10.83 ± 0.76^c^	11.96 ± 0.55^c^
* Salmonella enteritidis*	3.10 ± 0.10^a^	6.86 ± 0.71^b^	10.66 ± 0.57^c^	13.50 ± 0.50^d^	14.50 ± 0.50^d^
Gram-positive bacteria					
* Bacillus subtilis*	3.53 ± 1.45^a^	6.23 ± 1.09^b^	8.50 ± 0.50^c^	11.50 ± 1.04^d^	11.83 ± 0.76^d^
* Staphylococcus aureus*	3.30 ± 0.70^a^	8.13 ± 0.85^b^	10.66 ± 1.15^c^	18.83 ± 0.76^e^	15.83 ± 0.28^d^
Fungi					
* Aspergillus niger*	1.53 ± 0.45^a^	5.16 ± 0.96^b^	21.16 ± 1.04^d^	12.83 ± 0.76^c^	11.16 ± 0.76^c^
* Fusarium phyllophilum*	1.06 ± 0.20^a^	6.20 ± 0.91^b^	26.50 ± 0.50^e^	16.83 ± 0.76^d^	13.50 ± 0.50^c^

In each raw, data with the same superscript letter were not significantly different (p˃0.05), while those with different ones were significantly different (*p* < 0.05). Results were expressed as mean ± SD, data obtained from three experiments

## HPLC results

Although nineteen phenolic compounds were identified in the three grape seeds extracts, their concentrations were different and dependent on the type of solvent used in the extraction process. According to Table 4, the lowest concentration was observed with water extract followed by ethanolic extract then methanolic extract. Water extract contained high concentration of pyrogallol, benzoic acid, salicylic acid, ellagic acid, catechein, 4-hydroxy-3-methoxybenzoic acid and 4-Hydroxybenzoic acid (88.70, 36.93, 33.13, 25.16, 22.36, 19.13 and 18.60 ppm, respectively). Ethanolic extract contained high concentration of pyrogallol, gallic acid, salicylic acid, benzoic acid, ellagic acid, 4-hydroxybenzoic acid and 4-hydroxy-3-methoxybenzoic acid (416.66, 347.13, 200.96, 185.56, 130.46, 129.63 and 116.26 ppm, respectively). Methanolic extract contained high concentration of 4-hydroxybenzoic acid, pyrogallol and gallic acid (1471.13, 1129.83 and 850.50 ppm, respectively) followed by catechein, caffeine, benzoic acid and chlorogenic acid.

**Table 4:** Phenolic compounds in grape seeds extracts

Compound	Chemical Formula	Concentration (ppm)		
		Water extract	Ethanolic extract	Methanolic extract
Gallic acid	C_7_H_6_O_5_	5.16 ± 0.35^a^	347.13 ± 5.17^b^	850.50 ± 0.70^c^
Pyrogallol	C_6_H_6_O_3_	88.70 ± 1.80^a^	416.66 ± 4.04^b^	1129.83 ± 19.33^c^
4-aminobenzoic acid	C_7_H_7_NO_2_	2.16 ± 0.35^a^	13.60 ± 0.45^b^	165.96 ± 1.33^c^
Protocatechuic acid	C_7_H_6_O_4_	6.46 ± 0.25^a^	21.96 ± 1.10^b^	240.86 ± 1.96^c^
Chlorogenic acid	C_16_H_18_O_9_	8.96 ± 0.25^a^	25.03 ± 0.30^b^	360.56 ± 1.04^c^
Catechein	C_15_H_14_O_6_	22.36 ± 0.60^a^	56.73 ± 0.30^b^	564.60 ± 2.02^c^
Catechol	C_6_H_6_O_2_	5.16 ± 0.40^a^	46.56 ± 0.50^b^	171.56 ± 1.40^c^
Caffeine	C₈H₁₀N₄O₂	4.10 ± 0.36^a^	31.56 ± 0.40^b^	432.20 ± 0.88^c^
4-Hydroxybenzoic acid	C_7_H_6_O_3_	18.60 ± 0.40^a^	129.63 ± 0.70^b^	1471.13 ± 1.10^c^
Caffeic acid	C_9_H_8_O_4_	4.36 ± 0.55^a^	20.53 ± 0.45^b^	54.80 ± 0.62^c^
4-Hydroxy-3-methoxybenzoic acid	C_8_H_8_O_4_	19.13 ± 0.15^a^	116.26 ± 0.66^b^	339.56 ± 1.88^c^
p-Coumaric acid	C_9_H_8_O_3_	3.10 ± z0.26^a^	14.63 ± 0.45^b^	50.30 ± 0.85^c^
Ferulic acid	C_10_H_10_O_4_	1.50 ± 0.26^a^	14.56 ± 0.32^b^	22.73 ± 0.60^c^
Ellagic acid	C_14_H_6_O_8_	25.16 ± 0.20^a^	130.46 ± 1.51^b^	54.6 ± 0.90^c^
Benzoic acid	C₇H₆O₂	36.93 ± 0.50^a^	185.56 ± 0.56^b^	429.3 ± 0.85^c^
Salicylic acid	C₇H₆O₃	33.13 ± 0.90^a^	200.96 ± 0.65^b^	317.33 ± 2.00^c^
3, 4, 5-trimethoxycinnamic acid	C_12_H_14_O_5_	5.80 ± 0.26^a^	30.53 ± 0.45^b^	50.40 ± 1.20^c^
Coumarin	C_9_H_6_O_2_	1.60 ± 0.40^a^	8.56 ± 0.45^b^	29.70 ± 0.34^c^
Cinnamic acid	C_9_H_8_O_2_	0.80 ± 0.30^a^	2.03 ± 0.25^b^	6.90 ± 0.36^c^

In each raw, data with the same superscript letter were not significantly different (*p* > 0.05), while those with different ones were significantly different (*p* < 0.05). Results were expressed as mean ± SD, data obtained from three experiments

Nine flavonoid compounds were identified in the different grape seeds extracts. Their concentrations were dependent on the solvent used in the extraction process. A significant high flavonoids concentration was found in the methanolic extract followed by the ethanolic extract then by the water extract. Hesperidine was the dominant flavonoid compound in the three extract (59.36, 460.50 and 629.56 ppm for water, ethanolic and methanolic extract, respectively), which was followed by naringin (24.10, 250.13 and 570.23 ppm for water, ethanolic and methanolic extract, respectively) and rutin (22.06, 96.30 and 281.93 ppm for water, ethanolic and methanolic extract, respectively) (Table 5).

**Table 5:** flavonoids concentration in grape seed extracts

Compound	Chemical Formula	Concentration (ppm)		
		Water extract	Ethanolic extract	Methanolic extract
Naringin	C_27_H_32_O_14_	24.10 ± 0.88^a^	250.13 ± 7.65^b^	570.23 ± 0.87^c^
Naringenin	C_15_H_12_O_5_	0.26 ± 0.20^a^	2.16 ± 0.15^b^	6.76 ± 0.25^c^
Rutin	C_27_H_30_O_16_	22.06 ± 0.20^a^	96.30 ± 0.65^b^	281.93 ± 2.11^c^
Hesperidine	C_28_H_34_O_15_	59.36 ± 0.76^a^	460.50 ± 0.88^b^	629.56 ± 1.88^c^
Quercetrin	C_21_H_20_O_11_	2.53 ± 0.45^a^	60.30 ± 0.90^b^	222.16 ± 2.74^c^
Quercetin	C_15_H_10_O_7_	0.63 ± 0.15^a^	10.53 ± 0.30^b^	24.73 ± 0.55^c^
Hespirtin	C_16_H_14_O_6_	2.23 ± 0.32^a^	6.63 ± 0.47^b^	33.83 ± 0.47^c^
Kaempferol	C_15_H_10_O_6_	2.90 ± 0.36^a^	8.20 ± 0.20^b^	27.46 ± 0.50^c^
4′,5,7-trihydroxyflavone	C_15_H_10_O_5_	2.23 ± 0.20^a^	7.40 ± 0.45^b^	18.46 ± 0.47^c^

In each raw, data with the same superscript letter were not significantly different (p˃0.05), while those with different ones were significantly different (*p* < 0.05). Results were expressed as mean ± SD, data obtained from three experiments

## Discussion

Natural antioxidant compounds like polyphenols are abundant in the plant materials. The past twenty years have seen a number of studies about these compounds, including those related to the health (Yilmaz and Toledo 2004), plant isolation (Bucić-Kojic et al. 2007), and the identification of the polyphenolic compounds and its antioxidant properties (Villarreal-Lozoya et al. 2007). The waste products that results from food preparation (seeds, skins, leaves, stems, etc.) attracted special attention since they are an inexpensive natural antioxidant source that may be employed in the food, cosmetic and pharmaceutical industries. Given that grapes are one of the most widely grown fruits in the world [78 million tons annually according to the Food and Agriculture Organization (FAO)] (Madadian et al. 2022), a significant amount of solid waste (seeds) that is highly rich in bioactive polyphenols is left over after grapes processing (Spigno and De Faveri 2007). According to Nawaz et al*.* (2006), grape seeds comprise roughly 60–70% of all extractable grape bioactive compounds and account for 15% of the solid waste in the alcoholic beverages industry. These seeds are a great source of proanthocyanidin, which have anti-wrinkle, anti-cancer, anti- cardiovascular, and anti-viral effects. They, also, contain phenolic compounds like epicatechins, epicatechins gallate and procyanidins (Shi et al. 2003).

The separation of phenolic compounds from plants depends on their chemical composition, the method of extraction, the amount of sample, duration and the conditions of storage in addition to the existence of contaminants (Cao and Prior 1999). Plant phenolic extracts are invariably blends of various phenolic classes that exhibit solvent-selective solubility. According to Perva-Uzulanic et al*.* (2006), using an alcoholic solution yields satisfactory outcomes for the extraction procedure. It is necessary to find a solvent that is appropriate for extracting the phenolic compounds in the extract because they are frequently linked to other biomolecules such as amino acids, carbohydrates, aromatic compounds, pigments, fats, and inorganic chemicals. The inefficiency of acetone and water for the extraction of total phenols from grape seeds was proven by Jayaprakasha et al*.* (2001). Yilmaz and Toledo (2006) showed that acetone/water or ethanol/water performed better as solvents than either one alone. Additionally, the methanolic extract was superior for catechins, epicatechins, and epigallocatechins.

In this study, Muscat of Alexandria grape seeds were extracted for the collection of phenolic compounds and flavonoids. Grape seeds were cleaned, dried and crushed into fine powder then mixed, individually, with different solvents [distilled water, ≥ 95.0% absolute ethanol and absolute methanol (acetone free)]. The mixtures were kept at room temperature overnight. According to Zhang et al. (2018), for the solvent to spread throughout the sample, the sample particles' size needs to be tiny and the extraction process should be performed at room temperature to result in a larger yield. However, during the extraction process, an excessively high temperature may result in the discharge of volatile chemicals. Zwingelstein et al*.* (2020) reported that excessive durations cannot impact the extraction process because it reaches an equilibrium state at a certain point in time. Moreover, a reasonable solid to solvent ratio is ideal because a ratio that is too high could lengthen the extraction process. The choice of solvents in this study was due to many factors: 1- Phenolic compounds and flavonoids are commonly recovered from plant materials using polar solvents. 2- Ethanol is suitable for individuals' consumption and has a reputation for being an effective solvent for the extraction of polyphenols. 3- According to Dai and Mumper (2010), methanol is often more effective at extracting smaller-molecular-weight polyphenols. Our results showed the effectiveness of methanol followed by ethanol in the extraction process.

Our results showed that grape seeds contained high percent of carbohydrates (82.6 g/100 g), 2.86 g/100 g fat and 7.06 g/100 g protein, which accounted for an energy of 384.46 kcal/100 g. High content of calcium and magnesium and low content of iron, zinc were found in the seeds; in addition to neglected content of mercury. Moreover, HPLC determined nineteen phenolic compounds and nine flavonoid compounds of the three grape seeds extracts and the concentration of the resulted components were different and solvent dependent. Highest amount of phenolic compounds and flavonoids were observed in the methanolic extract followed by the ethanolic extract and water extract. The water extract had a high concentration of the chemicals, pyrogallol benzoic acid, salicylic acid, ellagic acid, catechein 4-hydroxy-3-methoxybenzoic acid and 4-Hydroxy benzoic acid. Ethanolic extract was found to have high concentration of pyrogallol, gallic acid, salicylic acid, benzoic acid, ellagic acid, 4-hydroxybenzoic acid and 4-hydroxy-3-methoxybenzoic acid. Methanolic extract contained high concentration of 4-hydroxybenzoic acid, pyrogallol and gallic acid followed by catechein, caffeine, benzoic acid and chlorogenic acid. Hesperidine was the dominant flavonoid compound in the three extract, which was followed by naringin and rutin.

According to Librán et al. (2013), the amount of phenolic substances in grape seeds differed according to the process of extraction, ranging from 4.58 to 28.06 mg gallic acid equivalent/g dry weight. Nageb (2015) assessed the total phenolic compounds of grape seeds and found out that this value ranged between 115.28 and 324.75 mg gallic acid/100 g dry weight. Casazza et al. (2010) showed that the highest content of total polyphenols was found in o-diphenols (108.3 mg gallic acid equivalent/g dry weight) and flavonoids (47.2 mg catechin equivalent/g dry weight) of grape seeds. Additionally, Rockenbach et al. (2011) noted that the total phenolic compounds content of grape pulp was 74.75 mg gallic acid equivalent/g dry weight; which was greater than those determined by Abdrabba and Hussein (2016), who observed that the total phenolic content of red grape seeds was 73.59 mg gallic acid equivalent/100 g dry weight.

The type of solvent used in the extraction process significantly affected the yield %, phenolic compounds and flavonoids content in the extract. The highest yield % was obtained with ethanol followed by methanol then distilled water. On the other hand, the highest flavonoids content was obtained from the methanolic grape seed extract followed by ethanolic extract then by water extract. Grape seeds ethanolic and methanolic extract contained the highest total phenolic compounds when compared to water extract. These results are attributed to: 1- Higher methanol extraction may be related to methanol's superior ability to penetrate the cell membranes, which allows for the instantaneous release of the contents of cells into the solvent and, ultimately, a higher net yield of the raw extract. Prior research on bioassays in differential solvent systems supports our findings of increased yield and consequent biological activities of methanolic extract (Singh et al. 2017). 2- The shorter methyl radical size in comparison to ethanol is the reason for the greater total phenolic content and total flavonoids content in the methanolic extract (Boeing et al. 2014). 3- Because methanol is more polar than other organic solvents, numerous investigations have found larger levels of total phenolic content and total flavonoids content in the plant extract in its methanolic form. According to Truong et al. (2019), the methanolic extracts of *Severinia buxifolia* had greater levels of phenolic compounds, flavonoids and alkaloids. Of course, a number of optimization elements, including retention duration, temperature, and chemical grade, have a significant impact on the extraction process's effectiveness (Turkmen et al. 2006).

According to Kumar et al. (2012), the phenolic compounds, flavonoids, and other related phytochemicals considerably lessen the harmful effects of reactive oxygen species (ROS), which are produced as a result of the oxidative stress and are responsible for a number of human diseases, including cancer, diabetes, heart disease, and degenerative disorders. Phenolic compounds have been demonstrated to be produced by plants in reaction to oxidative damage (Minhas et al. 2016). Conversely, the antioxidant properties of flavonoids, a type of phenolic chemicals, are widely recognized (Hernández et al. 2009). In addition to being antioxidants, flavonoids stabilize scavenging molecules that ROS flooding would otherwise wash away. Large amounts of free OH groups, particularly 3-OH, and the resulting increased reactivity of flavonoid hydroxyl groups with oxidative stress are attributed to this dual role (Nijveldt et al. 2001).

Our results showed that to isolate the phytochemicals that play the role of antioxidants within grape seeds, the most effective solvent, proved to be methanol or ethanol. In this study, the methanolic extract of grape seeds showed the highest antioxidant (558.30 µMol/g of Trolox equivalent) activity followed by the ethanolic extract (483.66 µMol/g of Trolox equivalent) then by the water extract (180.96 µMol/g of Trolox equivalent). As pointed out by Mohammedelnour et al. (2017), these findings imply that the alcoholic solvents are efficient in the extraction of the chemicals that give the grape seeds their antioxidant capacity. Grape seeds methanolic extract show better antioxidant activity than grape seeds ethanolic extract in terms of total polyphenols and flavonoids. Many works have been reported on the correlation between the antioxidant activity and the total phenolic content of plant extracts whose samples were prepared using different solvents (Bartolomé et al. 2004; Kedage et al. 2007; Jayaprakasha et al. 2008; Radovanovic et al*. *2009). The researchers also noted a strong correlation between the total phenolic compounds and the DPPH radical scavenging ability of plant samples that were treated with ethanol extract. The lowest contents of the antioxidant capacity were detected in grape seeds prepared by water extraction (198. 62 Mol Trolox equivalent/gm). As for extraction solvent, water was found to be unsuitable for the extraction in this case compared to pure acetone or ethanol as supported by Vayupharp and Laksanalamai (2012). Pinelo et al. (2005) also found that methanol solvent showed higher selectivity towards phenolic chemicals than water extracts and 96% ethanol. Compared with other 100% solvents, the 100% methanol extract was richer in the flavonoid compounds. Methanolic extract's strongest antioxidant activity suggests a connection to more polar components (Do et al. 2014). Keri and Patil (2014) showed that the polar chemicals include gallic acid, tannic acid and catechins. Ahmadi (2022) reported that several other secondary metabolites, including alkaloids, polyphenols, and carotenoids, have also been shown to exhibit strong antioxidant properties. The plant extract's higher antioxidant activity may also be related to other electron donors that can interact with free radicals and change them into more stable products leading to breaking of the chain of free radicals (Figueroa et al. 2014). Our findings were consistent with Foo et al. (2017) who showed a strong correlation between the total phenolic compounds and the antioxidant activity, as the total phenolic compounds is a major antioxidant present in a variety of medicinal plants (Lewis 2017). Also, Ahani and Attaran (2022) showed a strong antioxidant activity in the sea buckthorn methanolic extract. The oxidation/reduction properties of these phytochemicals serve as singlet oxygen inhibitors, hydrogen donors, reducing substances, and potential metal chelators, all of which enhance their antioxidant potential (Valentão et al. 2003). Phenolic compounds and their derivatives' hydroxyl groups stop the process of free radical production by reacting with the reactive nitrogen and oxygen species in a termination reaction (Heim et al. 2002).

In this study, no significance difference was observed in the hemolysis inhibition percent between the ethanolic and the methanolic extracts of grape seeds at 1000 μg/ml. On the other hand, the hemolysis inhibition activity of methanolic grape seeds extract was significantly higher than that of the ethanolic grape seeds extract at the remaining tested concentrations (100–800 µg/ml). Moreover, the aqueous grape seeds extract showed a significant low hemolysis inhibition % when compared to the ethanolic or methanolic grape seeds extract at all tested concentrations. These results were attributed to the phenolic compounds and flavonoids content in grape seeds. Our results were similar to those of Nageb (2015) who revealed that regarding their better polarity and higher solubility for phenolic compounds from plant materials, both methanol and ethanol yield higher efficiency in extracting phenolic compounds from the studied plant wastes than acetone and ethyl acetate. Additionally, several studies established that methanol was the most suitable extraction solvent for phenolic compounds from the different plant materials (Yu et al. 2006; Makris et al. 2007; Zulkifli et al. 2012; Mohammedelnour et al. 2017).

In this study, water extract showed the largest inhibition zone against the fungal strains (*Aspergillus niger* and *Fusarium phyllophilum*) indicating its powerful antifungal activity. Ethanolic extract showed high antibacterial activity against *Staphylococcus aureus* followed by the methanolic extract. No significance difference was observed in the antibacterial activity against *Escherichia* coli, *Salmonella enteritidis, Bacillus subtilis* and *Aspergillus niger* between the ethanolic and methanolic extracts. Based on the presence of numerous phytoconstituents which vary in their concentrations in grape seeds extracts, the observed antibacterial effect of the extract could be attributed by an additive and synergistic effect of the phytochemicals on the antimicrobial activity. Regarding the antimicrobial activity of the plant extracts, methanol was determined to be the most suitable solvent for the extraction of plant components. Aqueous and ethanol came in second and third, respectively, after methanol. The existence of distinct bioactive chemicals in test plants, the solvents' extraction capability, the sensitiveness of the microbial strains employed, and the combined impact of these factors were more likely to be the cause of the various antimicrobial properties exhibited by various plant extracts (Singh et al. 2017). The elevated quantity of total flavonoids content extracted by methanol, as revealed by the phytochemical analysis of the examined plants, may be the cause of the methanol extract's stronger antimicrobial action. It has been observed that the flavonoids hinder the metabolic pathways in bacteria, including the formation of nucleic acids (Cushnie and Lamb 2005). Flavonoids' impact on cellular membrane permeability is another factor contributing to their potent antibacterial action. Phenolic chemicals, alongside flavonoids, are crucial for preventing the growth of microbes. Phenols' C3 side chain reduces the amount of oxidation that gives them their antibacterial properties (Baba and Malik 2015). By inhibiting proteases and reacting with other amino acids and sugars, polyphenols' partial hydrophobicity further adds to microbial death (Pyla et al. 2010). Regarding the microorganisms’ response to the tested substances, it is crucial to highlight that the difference in the cell membranes’ morphological structure also applies to both gram-negative and gram-positive bacteria. This is even more valid in the case of gram negative bacteria owing to the fact that they have a distinct outer phospholipid membrane with different lipopolysaccharide structures making their surfaces extremely hydrophilic (Daoud et al. 2019). However, the fact that gram positive bacteria are only enveloped in peptidoglycan layer and hence makes the bacteria easy for hydrophobic substances to penetrate makes it vulnerable to the drugs. According to Mehmood et al. (2022), the plant extract had a greater effect on gram-positive *Staphylococcus aureus* than gram-negative *Escherichia coli*. This is explained by the fact that gram-negative bacteria have a unique outer barrier that keeps extracellular material from entering the cell, but gram-positive bacteria do not. Kumar and Vijayalakshmi (2011) found that linoleic acid was one of the components present in the grape seeds extract; Jayaprakasha et al. (2001) added that linoleic acid showed antioxidant and antibacterial activities. Stojkoviæ et al. (2013) explained the antibacterial, inflammatory and antioxidant properties of grape seeds by the presence of gallic and caffeic acids in their extract.

However, the activity and phytochemicals content in plant extracts differ from a research to another due to many reasons such as the variations in the plant matrices, extraction procedure and conditions (temperature and duration) (Robards et al*.* 2003). Also, the variations in the extraction solvents led to variations in the extracts' compositions and antioxidant properties (Pinelo et al*.* 2024). An extract with a phenolic component with a greater number of hydroxyl groups has an enhanced antioxidant capacity (Arabshahi-D et al. 2007). In conclusion, grape seeds contained several bioactive compounds that exerted an antioxidant, hemolysis inhibition and anti-microbial activities. These activities depends on the concentration of phenolic compounds and flavonoids in the grape seeds extracts. Hence, the extraction solvents would have to be composed of the right solvents to ensure the extraction of fractions with high levels of polyphenols. Methanol was the superior solvent in the extraction of high percent of these phytochemicals followed by ethanol. On the other hand, water was not efficient in the extraction of phenolic compounds and flavonoids due to their insolubility in water (Koffi et al*.*^2010^). The investigated seed extract has a lot of potential for use in the food industry as a source of bioactive chemicals, such as phenolic compounds for functional meals or dietary supplements (Schreibe et al. 2013).

## Data Availability

All data generated or analysed during this study are available from the corresponding author on a reasonable request.

## Abbreviations

DPPH: 1,1-Diphenyl-2-picryl hydrazyl
A.O.A.C.: Association of Official Analytical Chemists
FAO: Food and Agriculture Organization
FDA: Food and Drug Administration
GRAS: Generally recognized as safe
HPLC: High performance liquid chromatography
IZ: Inhibition zone
NOEAL: No observed-adverse effect level
ROS: Reactive oxygen species
SD: Standard deviation
TEAC: Trolox equivalent antioxidant capacity
